# Portable Smartphone-Based Thermal Imaging for Real-Time Assessment of Coronary Artery Bypass Grafting Graft Patency and Cardioplegia Distribution: A Feasibility Case Series

**DOI:** 10.21470/1678-9741-2025-0328

**Published:** 2026-02-18

**Authors:** Henrique Madureira da Rocha Coutinho, Eduardo Saito, Gustavo Kikuta, Bernardo Ferreira Americano Brasil, Pedro Ricardo Garcia Jazbik, Gabriel Bittencourt, Giovana Pedro, Nathalia Lino, Joaquim Henrique de Souza Coutinho, Rodolfo Acatauassú Nunes

**Affiliations:** 1 Department of Cardiovascular Surgery, Hospital Universitário Pedro Ernesto, Universidade do Estado do Rio de Janeiro (HUPE-UERJ), Rio de Janeiro, Rio de Janeiro, Brazil; 2 Department of Cardiovascular Surgery, Hospital das Clínicas de Teresópolis Constantino Ottaviano, Centro Universitário da Serra dos Órgãos (HCTCO-UNIFESO), Teresópolis, Rio de Janeiro, Brazil; 3 Department of Thoracic Surgery, Hospital Universitário Pedro Ernesto, Universidade do Estado do Rio de Janeiro (HUPE-UERJ), Rio de Janeiro, Rio de Janeiro, Brazil

**Keywords:** Coronary Artery Bypass, Thermography, Cardioplegia, Blood Vessel Patency, Intraoperative Monitoring.

## Abstract

**Introduction:**

Intraoperative assessment of graft patency and cardioplegia distribution during coronary artery bypass grafting (CABG) is essential for surgical success but remains challenging in resource-limited settings. Conventional tools such as flow measurement or intraoperative angiography are often unavailable in public hospitals, where evaluation relies mainly on clinical judgment.

**Methods:**

We conducted a prospective observational case series of 10 CABG patients operated between February and July 2025 at a public university hospital. Myocardial temperature distribution during cardioplegia infusion and after grafting was monitored with a portable smartphone-based thermal camera (FLIR One Pro).

**Results:**

Thermal imaging documented cardioplegia distribution and graft patency in all cases. In one patient, heterogeneous distribution in the lateral wall was identified, leading to a change in surgical sequence and improved myocardial protection. All patients were weaned from bypass with stable rhythm, showed expected postoperative troponin kinetics, and had no new wall motion abnormalities on echocardiography.

**Conclusion:**

Portable thermal imaging is a feasible, safe, and inexpensive method for real-time intraoperative evaluation of cardioplegia distribution and graft patency in CABG. It may represent a valuable adjunct in resource-limited environments. Larger studies are needed to confirm these findings.

## INTRODUCTION

**Table:** 

Abbreviations, Acronyms & Symbols	
CABG	= Coronary artery bypass grafting
EuroSCORE	= European System for Cardiac Operative Risk Evaluation
HUPE-UERJ	= Hospital Universitário Pedro Ernesto of the Universidade do Estado do Rio de Janeiro
IQR	= Interquartile range
IRT	= Infrared thermography
LAD	= Left anterior descending artery
SD	= Standard deviation
TTFM	= Transit-time flow measurement

Coronary artery bypass grafting (CABG) remains the gold standard for the treatment of complex multivessel coronary artery disease, providing symptomatic relief and survival benefit compared with medical therapy or percutaneous strategies^[1,2]^. Long-term outcomes are strongly dependent on graft patency, making intraoperative assessment of coronary anastomoses a critical factor for surgical success^[3,4]^.

Several methods have been described for intraoperative evaluation of graft function and myocardial protection, including intraoperative angiography, electromagnetic flowmetry, and transit-time flow measurement (TTFM)^[5-7]^. Although these techniques provide valuable information, they are often costly, invasive, and not universally available, particularly in hospitals with limited resources^[8,9]^.

Early studies explored the use of infrared thermography (IRT) as a tool for visualizing myocardial perfusion and cardioplegia distribution^[10,11]^. Experimental and clinical investigations confirmed that thermal imaging could detect temperature changes in real time, correlating with regional blood flow^[12,13]^. Subsequent developments expanded its applications to the intraoperative evaluation of bypass grafts and myocardial bridges^[14]^.

More recent research has reinforced the potential of IRT as a low-cost and non-invasive imaging modality, particularly with the advent of portable, smartphone-based devices that allow easy integration into the surgical workflow^[15]^. These tools may overcome the limitations of conventional technologies, offering an accessible method for real-time assessment of graft patency and cardioplegia distribution.

Given these considerations, the present study reports our initial experience with portable thermography during CABG, aiming to demonstrate its feasibility as a practical intraoperative monitoring tool.

## METHODS

This prospective, observational case series was conducted at the Hospital Universitário Pedro Ernesto of the Universidade do Estado do Rio de Janeiro (HUPE-UERJ), a public institution exclusively serving the Brazilian Unified Health System (Sistema Único de Saúde or SUS). The study protocol followed the principles of the Declaration of Helsinki and was approved by the local Research Ethics Committee (approval number 94126624.1.0000.5259). Consecutive patients undergoing elective CABG between February 2025 and July 2025 were included. Eligibility criteria comprised patients older than 18 years, with a formal indication for isolated CABG, who provided informed consent to participate. Patients with intraoperative hemodynamic instability or in whom adequate imaging could not be obtained were excluded.

**Fig. 1:**
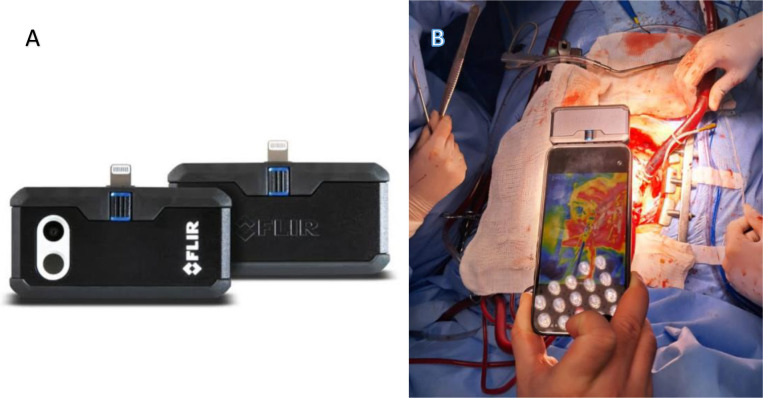
A) and B) Portable thermal camera (FLIR One Pro) attached to a smartphone, used for intraoperative acquisition of thermographic images at approximately 30 cm from the surgical field.

All surgeries were performed through median sternotomy with the use of cardiopulmonary bypass and administration of cold blood cardioplegia delivered exclusively in an anterograde fashion. The number of grafts varied according to coronary anatomy and individualized revascularization strategies. After completion of each distal anastomosis, and again at the end of cardiopulmonary bypass, thermal images were acquired using the portable FLIR One Pro camera (FLIR Systems, United States of America), coupled to a smartphone. The device was positioned approximately 30 cm above the cardiac surface, and images were continuously recorded (Figure 1).

Thermal images were analyzed in real time by the surgical team, with particular attention to the distribution of the thermal gradient following cardioplegia administration. Homogeneous patterns of myocardial warming were interpreted as adequate perfusion, while heterogeneous or delayed thermal patterns were suggestive of technical issues with the grafts. The primary endpoint was the feasibility of intraoperative image acquisition and interpretation using portable thermography. Secondary endpoints included the assessment of cardioplegia distribution, concordance between the surgeon’s clinical judgment and thermographic findings, and the additional time required for image acquisition.

Given the exploratory nature of this case series, no formal sample size calculation was performed. Data were analyzed descriptively. Continuous variables are presented as median and interquartile range, and categorical variables as absolute and relative frequencies.

## RESULTS

**Fig. 2:**
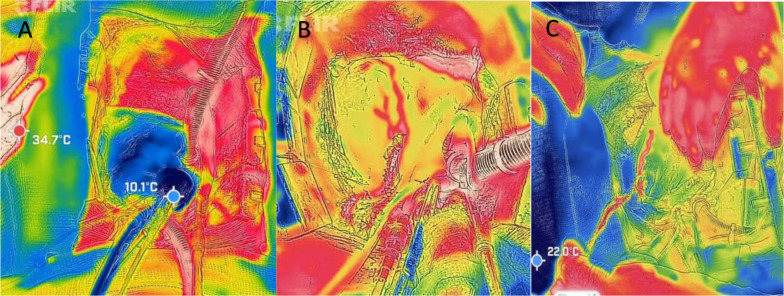
A) Assessment of homogeneous distribution of cold cardioplegia, resulting in the entire heart appearing blue (cold color). B) Evaluation of the left internal thoracic artery graft to the left anterior descending artery (LAD), showing complete filling of the LAD and opacification of a diagonal branch. C) Assessment of the saphenous vein graft to the obtuse marginal artery using warmed saline solution to provide contrast.

**Fig. 3:**
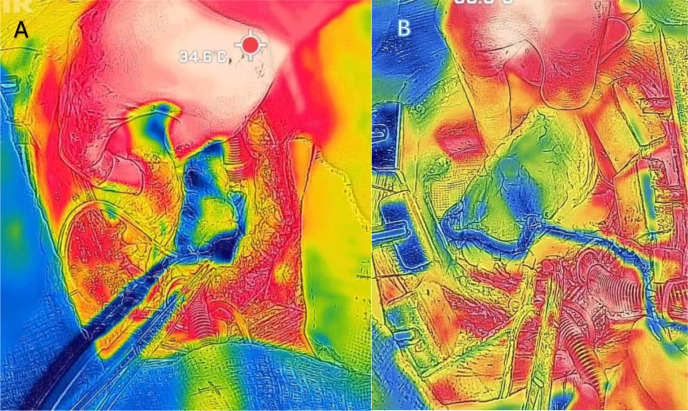
A) Heterogeneous distribution of cardioplegia, with persistence of a warmed lateral wall (reddish color) after manual rotation of the heart. B) Sequential saphenous vein grafting to two obtuse marginal arteries, with infusion of cold cardioplegia through the graft.

All ten patients underwent intraoperative thermal imaging monitoring during CABG procedures (Figure 2). Baseline demographic and clinical characteristics of the cohort are presented in Table 1. In nine cases, cardioplegia distribution was homogeneous across the myocardial territories. In one patient, however, heterogeneous distribution was observed in the lateral wall during the initial infusion (Figure 3). This prompted a modification of the surgical strategy: instead of following the institutional routine - right coronary system first, then lateral wall grafts, and finally the left internal mammary artery to the left anterior descending artery - the obtuse marginal artery was grafted first. A saphenous vein graft was anastomosed to the obtuse marginal, and cardioplegia was subsequently infused through this conduit, resulting in improved distribution in the lateral wall territory.

**Table 1:** Baseline demographic and clinical characteristics.

Variable	Mean ± SD	Median (IQR)	Range
Age (years)	61.8 ± 6.7	62 (57 - 67)	51 - 72
Male sex	8 (80%)	-	-
Body mass index (kg/m^2^)	27.3 ± 2.9	27 (25 - 29)	23 - 32
Left ventricular ejection fraction (%)	55.2 ± 7.1	55 (50 - 60)	45 - 68
EuroSCORE II (%)	2.1 ± 0.9	2.0 (1.5 - 2.5)	1.0 - 4.0

EuroSCORE=European System for Cardiac Operative Risk Evaluation

Values are expressed as mean ± standard deviation (SD), median (interquartile range [IQR]), or number (percentage), as appropriate

All patients were successfully weaned from cardiopulmonary bypass with spontaneous and regular sinus rhythm. Postoperative electrocardiograms showed no new ischemic changes. Troponin levels in the immediate postoperative period were consistent with typical values for CABG and demonstrated a rapid decline in the subsequent days. Patient comorbidities, including hypertension, dyslipidemia, and diabetes mellitus are summarized in Table 2. Transthoracic echocardiography performed postoperatively revealed no new regional wall motion abnormalities and preserved left ventricular systolic function compared to baseline. Intraoperative variables, such as the number of distal anastomoses, use of conduits, cardiopulmonary bypass and cross-clamping times, and total cardioplegia volume, are detailed in Table 3.

**Table 2:** Patient comorbidities.

Comorbidity	n (%)
Hypertension	6 (60%)
Dyslipidemia	4 (40%)
Diabetes mellitus	3 (30%)
Smoking (current or former)	3 (30%)
Peripheral arterial disease	1 (10%)
Prior cerebrovascular disease	1 (10%)
Hypothyroidism	1 (10%)

Data are presented as number of patients (percentage). Percentages were calculated considering the total cohort of 10 patients

**Table 3:** Intraoperative data.

Variable	Mean ± SD	Median (IQR)	Range
Number of distal anastomoses	3.2 ± 1.1	3 (2 - 4)	2 - 5
Use of left internal thoracic artery	10 (100%)	-	-
Additional saphenous vein grafts	7 (70%)	-	-
Aortic cross-clamping time (min)	61.4 ± 11.2	62 (50 - 70)	43 - 70
Cardiopulmonary bypass time (min)	74.1 ± 15.5	72 (65 - 85)	50 - 95
Total cardioplegia volume (mL)	196 ± 45	190 (170 - 220)	130 - 280
Thermography applied	10 (100%)	-	-
Documented cardioplegia distribution	10 (100%)	-	-

Values are expressed as mean ± standard deviation (SD), median (interquartile range [IQR]), or number (percentage), as appropriate

## DISCUSSION

The assessment of graft patency during CABG remains a challenge in many centers, particularly in hospitals serving exclusively the public health system, where advanced technologies such as TTFM or intraoperative angiography are not routinely available. In such settings, the evaluation of graft function relies almost exclusively on the surgeon’s clinical judgment. This limitation underscores the need for accessible, reliable, and non-invasive tools to support intraoperative decision-making.

Thermal imaging has emerged as a promising adjunct in cardiovascular surgery, providing real-time assessment of myocardial perfusion and graft patency without the need for invasive instrumentation^[1-4]^. The development of compact, portable cameras, such as the FLIR One Pro, allows integration of this technology into routine surgical workflow with minimal training requirements and without significant additional costs. Unlike conventional imaging modalities, thermal cameras offer an immediate and intuitive visualization of temperature changes, which can reflect the distribution of cardioplegia and, indirectly, the adequacy of graft perfusion^[5-7]^.

In the present case series, thermal imaging enabled continuous monitoring of myocardial temperature during cardioplegia infusion and graft construction. Importantly, in one patient, heterogeneous distribution in the lateral wall was detected, leading to a modification of the surgical sequence with adequate homogeneous myocardial protection. This illustrates the potential of thermal imaging not only as a confirmatory tool but also as a real-time guide for intraoperative decision-making. These findings are consistent with previous reports where thermal imaging supported adjustments in myocardial revascularization strategies and improved the understanding of perfusion patterns^[8-10]^.

When compared with established intraoperative tools, thermal imaging offers clear advantages in cost and availability. While TTFM provides quantitative flow data and indocyanine green angiography allows direct visualization of graft patency, both methods require specialized equipment and consumables, limiting their use in resource-constrained environments^[11-13]^. In contrast, thermal imaging is inexpensive, portable, and easy to apply, though it provides indirect rather than quantitative assessment of flow. This makes it a valuable complementary tool, particularly in hospitals where more sophisticated modalities are not feasible.

The advantages of this technology are particularly relevant in low-resource environments. The FLIR One Pro is a portable and relatively inexpensive device compared to other intraoperative assessment modalities, making it accessible to hospitals serving only the public health system. Its ease of use, compact design, and adequate image resolution make it feasible for integration into daily clinical practice without significant infrastructure investment^[14]^.

### Limitations

Nevertheless, several limitations should be acknowledged. Thermal imaging provides indirect evidence of perfusion based on surface temperature changes and cannot replace gold-standard flow measurements or angiographic confirmation^[15]^. Furthermore, image interpretation may be affected by factors such as ambient temperature, surgical lighting, and tissue exposure. The present series is also limited by its small sample size and the absence of a comparator group, which precludes stronger conclusions. However, this reflects the beginning of our clinical experience with this technology. We considered it important to publish these initial cases as a pilot study, since the early results appear promising and the technique is technically simple, which may encourage other centers to adopt similar approaches.

## CONCLUSION

This preliminary case series demonstrates that intraoperative thermal imaging using a portable device is feasible and safe for assessing both cardioplegia distribution and graft patency during CABG. The technology proved useful for detecting perfusion heterogeneity and guiding intraoperative decisions, even in a resource-limited public hospital setting where advanced flow measurement tools are not available. Its portability, ease of use, and low cost make it an attractive adjunct to standard surgical practice. Although promising, these findings must be interpreted with caution, as the study is in its initial phase. Further research with a larger cohort and extended follow-up is warranted to validate its clinical utility and define its role in routine cardiac surgery.

## Data Availability

The authors declare that the data supporting the findings of this study are available within the article.

## Artificial Intelligence Usage

The authors declare use of ChatGPT5.0 for text editing and translation from Portuguese to English. The content produced by the artificial intelligence tool was revised and edited by the authors as necessary, and they take full responsibility for the content to be published.

## References

[1] Mohr FW, Falk V, Philippi A, Autschbach R, Krieger H, Diegeler A, Dalichau H. (1994). Intraoperative assessment of internal mammary artery bypass graft
patency by thermal coronary angiography. Cardiovasc Surg.

[2] Szabo T, Fazekas L, Horkay F, Geller L, Gyongy T, Juhasz-Nagy A. (1999). Intraoperative IR imaging in the cardiac operating
room. In: Proc SPIE.

[3] Balacumaraswami L, Abu-Omar Y, Choudhary B, Pigott D, Taggart DP. (2005). A comparison of transit-time flow measurement and intraoperative
fluorescence imaging for assessing coronary artery bypass graft
patency. J Thorac Cardiovasc Surg.

[4] Balacumaraswami L, Taggart DP. (2007). Intraoperative imaging techniques to assess coronary artery
bypass graft patency. Ann Thorac Surg.

[5] Taggart DP, Choudhary B, Anastasiadis K, Abu-Omar Y, Balacumaraswami L, Pigott DW. (2003). Preliminary experience with a novel intraoperative fluorescence
imaging technique to assess the patency of bypass grafts in coronary artery
surgery. Ann Thorac Surg.

[6] Desai ND, Miwa S, Kodama D, Koyama T, Cohen G, Christakis GT (2006). A randomized comparison of intraoperative indocyanine green
angiography and transit-time flow measurement to detect technical errors in
coronary bypass grafts. J Thorac Cardiovasc Surg.

[7] Di Giammarco G, Pano M, Cirmeni S, Pelini P, Vitolla G, Di Mauro M. (2006). Predictive value of intraoperative transit-time flow measurement
for short-term graft patency in coronary surgery. J Thorac Cardiovasc Surg.

[8] Kieser TM, Rose S, Kowalewski R, Belenkie I. (2010). Transit-time flow predicts outcomes in coronary artery bypass
graft patients: a series of 1000 consecutive arterial grafts. Eur J Cardiothorac Surg.

[9] Zeng C, Li X, Dai Y, Zhou Y, Li C, Liu N, Wang J. (2021). Transit time flow measurement predicts graft patency in off-pump
coronary artery bypass grafting upon 5-year angiographic
follow-up. J Cardiothorac Surg.

[10] Hol PK, Lingaas PS, Lundblad R, Rein KA, Vatne K, Smith HJ (2004). Intraoperative angiography leads to graft revision in coronary
artery bypass surgery. Ann Thorac Surg.

[11] Desai ND, Miwa S, Kodama D, Cohen G, Christakis GT, Goldman BS (2005). Improving the quality of coronary bypass surgery with
intraoperative angiography: validation of a new technique. J Am Coll Cardiol.

[12] Tolegenuly A, Ordiene R, Jakuska P, Mamedov A, Unikas R, Benetis R. (2022). Intraoperative angiography during coronary artery bypass
grafting. Perfusion.

[13] Nazer RI, Mobeirek AF, Alharbi WM, Albarrati AM. (2025). Thermal Imaging-Guided Surgical Unroofing of a Myocardial Bridge
in a Patient With Refractory Angina. JACC Case Rep.

[14] Crisi G, Filice S, Scoditti U. (2019). Arterial spin labeling MRI to measure cerebral blood flow in
untreated ischemic stroke. J Neuroimaging.

[15] Di Giammarco G, Marinelli D, Foschi M, Di Mauro M. (2017). Intraoperative graft verification in coronary
surgery. J Cardiovasc Med (Hagerstown).

